# Establishment of comparative performance criteria for IVF centers: correlation of live birth rates in autologous and donor oocyte IVF cycles

**DOI:** 10.1186/1477-7827-12-122

**Published:** 2014-12-04

**Authors:** Vitaly A Kushnir, Pallavi Khanna, David H Barad, Norbert Gleicher

**Affiliations:** The Center for Human Reproduction (CHR), 21 East 69th Street, New York, NY 10021 USA; Icahn School of Medicine at Mount Sinai (Jamaica) Program, Queens Hospital Center, Jamaica, NY USA; The Foundation for Reproductive Medicine, New York, NY USA

**Keywords:** Assisted reproductive technology (ART), In vitro fertilization (IVF), Live birth rates, Donor oocyte cycles, Quality controls

## Abstract

**Background:**

To assess whether an objective performance criterion for in vitro fertilization (IVF) centers can be established.

**Methods:**

A retrospective analysis of 2011 National ART Surveillance System data for 451 U.S. IVF centers, 137 of which were included in the analysis since they performed >20 fresh embryo transfers per age group and >20 fresh oocyte donor transfers. The analysis of autologous cycles was restricted to women under age 40. The main outcome measure was correlation between center-specific live birth rates (LBR) in autologous and donor oocyte cycles.

**Results:**

55.6% donor and 46.7%, 39.1% and 28.7% (for ages <35, 35–37 and 38–40 years) autologous cycles resulted in live births per fresh embryo transfer. Donor LBR predicted autologous LBR (< 35 years, P < 0.001; 35 – 38 years, P < 0.001; 38 – 40 years, P = 0.015). Clinics with high prevalence of patients with diminished ovarian reserve had lower autologous LBR per age group (P = 0.015). Every 10% increase in donor LBR increased odds of autologous LBR above the age-adjusted national average by 68% (OR 1.68; 95% CI 1.36 – 2.07; P < 0.001).

**Conclusions:**

Since center-specific donor and autologous IVF cycle outcomes correlate, and as donor cycles reflect fewer patient covariates, they represent a first comparable performance measure between centers, allowing for internal as well as external quality control.

## Background

Center-specific national reports of IVF cycle outcomes, published by the Centers for Disease Control and Prevention (CDC) and the Society for Assisted Reproductive Technology (SART), allow patients limited insights into center performances. Both reports, however, emphasize in introductory materials that center-specific outcomes should not be used to directly compare centers since severity of infertility in treated patient populations can greatly vary [1, 2].

Interest in clinical outcome measures, which allow objective clinical performance comparisons have been increasing in the general public, among medical insurance companies and from government agencies [3]. The two currently existing national reporting systems largely focus on pregnancy and delivery rates, indiscriminate of patient characteristics, though the focus is shifting to also include measures emphasizing favorable neonatal/perinatal outcomes [4–6].

An optimal reporting system would account for inherent differences in patient populations, patient selection biases, clinical IVF protocols and laboratory procedures as well as neonatal and perinatal outcomes. To directly compare qualities of IVF programs accurately, assessment measures would, therefore, have to account for all of these covariates, a highly complex and currently unachievable goal.

Most clinical IVF programs offer in parallel autologous programs, which utilize the patients’ own oocytes, and programs, which utilize oocytes from mostly young oocyte donors, usually under age 30 years, and with normal ovarian reserve. In contrast to autologous IVF cycles, donor oocyte cycles, therefore, reflect fewer covariates, as age and ovarian reserve of oocyte donors can be assumed to be quite uniform between centers, while still maintaining the important variability of clinical cycle management and IVF laboratory performance, both very basic quality parameters for IVF centers.

In a first step, attempting to establish a model to compare performance criteria between IVF centers we assessed whether in individual programs center-specific live birth rates (LBR) in donor oocyte cycles, in principle, correlate with center-specific LBR in autologous IVF cycles. Assuming such a correlation could be confirmed, deviations in individual centers could then be used for internal as well as external quality control.

## Methods

We reviewed center-specific outcome data from U.S. National Assisted Reproductive Technology Surveillance System (NASS) reported by 451 centers for IVF cycles initiated in 2011 (non-donor and donor oocytes), which progressed to fresh embryo transfers [1]. Only clinics performing more than 20 fresh transfers from autologous oocytes per age group in women up to age 40 and more than 20 fresh oocyte donation cycles were included in order to focus on statistically meaningful LBR data. Clinics performing few cycles were excluded from analysis because very high or low LBR can be achieved in a given patient group by chance and do not reflect clinical or embryology laboratory management.

Outcome data reported in the NASS system for patients utilizing non-donor oocytes, going forward here described as autologous cycles, are stratified by female ages <35, 35–37 and 38–40 years.

One-hundred-and-thirty-one (131) centers were analyzed for female age <35, 124 centers for the 35–37 age group, and 119 centers for ages 38–40. Since most of the included clinics overlapped a total of 137 clinics were analyzed. LBR resulting from fresh embryo transfers from donor oocytes in those centers served as the predictor variable.

Since donor cycles usually involve only carefully selected, young and healthy donors, they remove significant patient covariates from consideration, which in autologous IVF cycles can greatly differentiate treated patient populations at different centers. Yet, at the same time, oocyte donor cycles are still subject to variations in clinical management and embryology laboratory performance between centers.

Donor IVF cycle outcomes, therefore, should approximate best possible LBR for all centers. Despite important potential limitations, including directed oocyte donations involving older donors and endometrial receptivity issues [7], both decreasing LBR, donor oocyte cycle outcomes should reflect center performance criteria more accurately than autologous cycle outcomes.

### Statistical analysis

LBR per fresh transfer was tabulated for each age group and for donor transfers in each included clinic. Comparisons of groups utilized logistic regression with a bivariate outcome variables, coded 1 for above average LBR for an age group and 0 for lower than average LBR. The actual donor LBR per center was considered the predictor variable. These results were then adjusted for the percent of patients with a diagnosis of diminished ovarian reserve (DOR) reported by each center. DOR, which diminishes LBR at all ages [8], was controlled for because unlike other common infertility diagnosis it is not readily overcome in IVF and thus helps to control for severity of patient populations across clinics [6]. Of all the infertility diagnosis tracked by the CDC, DOR is one of the most prevalent (Table 1) yet it is associated with the lowest IVF pregnancy rates. The statistical significance levels for all tests were set at 0.05. All statistical analyses were performed using SPSS Statistics version 21.

**Table 1:** **Percentage distribution of patient and treatment factors at all ART clinics and those included in the analysis (NASS data, year 2011)**

	All ART clinics (n = 451)		Included ART clinics (n = 137)	
	Median	Mode	Median	Mode
**Patient diagnosis**				
*Tubal factor*	15%	12%	13%	11%
*Ovulatory dysfunction*	15%	11%	12%	11%
*Diminished Ovarian Reserve*	28%	32%	29%	32%
*Endometriosis*	9%	5%	8%	5%
*Uterine factor*	4%	0%	4%	3%
*Male factor*	36%	32%	32%	26%
*Other factor*	9%	0%	12%	7%
*Unknown factor*	8%	0%	9%	2%
*Female factors only*	10%	10%	10%	8%
*Female and Male factors*	18%	11%	15%	14%
**Treatment factors**				
*Type of ART Procedural factor (with ICSI)*	72%	85%	70%	94%
*Percentage of Fresh nondonor cycles used a gestational carrier*	0%	0%	1%	1%
*Percentage of Fresh nondonor cycles with PGD performed*	3%	0%	4%	4%
*Percentage of Fresh nondonor cycles with elective single embryo transfers*	4%	0%	5%	2%
*Number of embryos transferred in nondonor oocyte cycles, female age <35 years*	2.0	2.0	1.9	2.0
*Number of fresh embryos transferred in donor oocyte cycles*	2.0	2.0	2.0	2.0

-No significant differences were found.

## Results

Table 1 demonstrates that for this study selected IVF centers did not differ from all reporting IVF centers in patient characteristics.

Among the analyzed IVF centers, the average donor cycle LBR per fresh transfer was 55.6% (95% CI 53.7 to 57.6). The LBR per fresh transfer in autologous IVF cycles was 46.7% (95% CI 45.1 – 48.4) for women <35 years, 39.1% (37.4 – 40.9) for the 35–37 age group and 28.7% (27.1 – 30.3) for women at ages 38–40 years. Binary logistic regression analysis of center-specific donor LBR with autologous center-specific LBR as the dependent variable, adjusted for the center’s percentage of women with DOR and for age (group) demonstrated that increasing percentages of DOR patients significantly decreased odds of having above average autologous IVF LBR (P = 0.015). Every 10% increase in a center’s donor LBR increased the center’s odds of having an autologous IVF cycle LBR above the age-adjusted national average by 68% (OR 1.68; 95% CI 1.36 – 2.07; P < 0.001).

Since donor LBR and patient age groups significantly interacted (P = 0.035), we also investigated separate models for each age group. As Table 2 demonstrates, odds ratios for each age group remained significant in themselves.

**Table 2:** **Binary logistic regression of above average autologous age specific oocyte LBR and donor oocyte LBR, adjusted for diagnosis of diminished ovarian reserve**

Age group	Average LBR per fresh transfer	p- value	Odds ratio	95% confidence interval
**Donor**	55.6%	--	--	--
**<35**	46.7%	0.027	1.45	1.04 – 2.02
**35-37**	39.1%	0.001	2.21	1.46 – 3.34
**38-40**	28.7%	0.016	1.56	1.08 – 2.24

**Figure 1:**
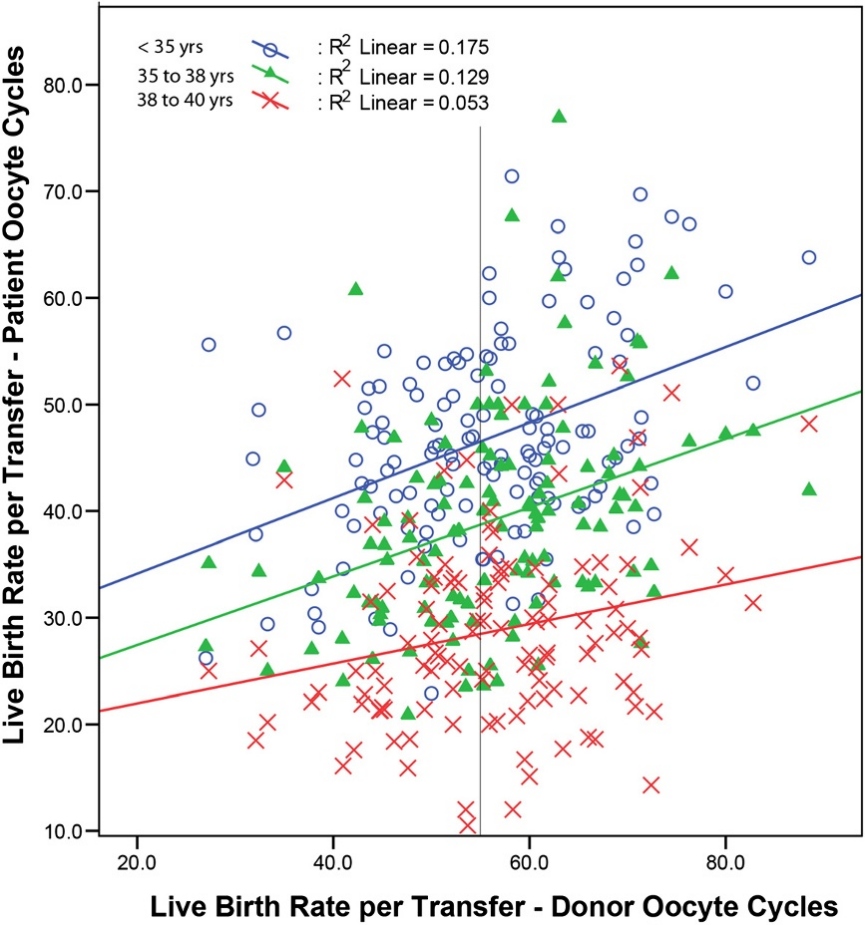
**Clinic specific autologous oocyte LBR compared to donor oocyte LBR in 2011.**

Figure 1 depicts the linear regression curves for the three age groups. Regression of autologous LBR against donor LBR, adjusted for percent DOR was also independently significant for all three age groups (<35 years, R^2^ = 0.18, F(2,118) = 14.9, P < 0.001; 35 – 38 years, R^2^ = 0.13, F(2,121) = 11.7, P < 0.001; 38 – 40 years, R^2^ = 0.05, F(2,116) = 7.13, P = 0.001).

## Discussion

As stated by administrative bodies of both currently existing U.S. national reporting systems for IVF cycle outcomes, they should not be used to compare individual centers [1, 2] because it is widely recognized that outcomes greatly vary based on prognostic criteria of treated patient populations. Yet, lay public, medical insurance companies and even government organizations do exactly that to select providers [3]. It, therefore, is not surprising that interest is growing in developing performance measures that would allow for a more reliable comparison between IVF centers.

The utilization of oocyte donation cycles in the U.S. has in recent years rapidly increased [9]. Most IVF centers in the country, therefore, now do offer donor egg cycles to their patients. Since egg donor selection in such cycles, largely, has to follow strict federal rules [10] and professional guidelines [11], donor/recipient cycles are subject to fewer oocyte- and ovarian reserve- associated covariates determining cycle outcomes than autologous cycles. Yet, donor/recipient cycles, nevertheless, are still subject to very important center-specific quality parameters, such as clinical management skills and embryology laboratory performance. Donor egg cycles, therefore, should represent a better performance measure than currently widely used autologous outcome reports to compare the quality of care among IVF centers. By demonstrating that center-specific LBR of donor and autologous cycles, indeed, statistically to a high degree correlate, and that this, independent of age, is true up to age 40 years, this study offers evidence that this hypothesis holds up clinically. Interestingly, this correlation was strongest at middle age, 35–37 years (Table 2), and Figure 1, also as one would expect, demonstrates a progressive decrease of the slope of regression with increasing female age group. This observation suggests that with increasing female age clinic/embryology laboratory performance plays less of a role in achieving success with autologous oocytes.

The validity of here proposed concept is further supported by the also expected observations that the prevalence of DOR in autologous cycles is statistically associated with center-specific LBR. DOR is well recognized to diminish LBR at all ages [8]. DOR diagnosis was reported for individual patients and therefore for their patient population by the fertility centers themselves; the accuracy of this diagnosis is currently not validated by the CDC via ovarian reserve parameters or number of retrieved oocytes.

Center specific LBR in donor/recipient cycles, thus, appear to represent a first tool to compare the clinical performance of IVF centers more objectively. It, however, represents only a first step, as currently nationally reported donor cycles still do include atypical cases, mostly involving directed (“open”) egg donation, where standard donor selection criteria often are waved by recipients and, therefore, older women serve as egg donors [7]. A center that does disproportionally many of such directed donor cycles, therefore, would be at a disadvantage. Furthermore, clinics which routinely practice single embryo transfer may be expected to have relatively lower LBR in both autologous and donor IVF cycles; however, such practice in the USA is still uncommon as seen in Table 1, with most IVF cycles resulting in transfer of at least two embryos.

While IVF success is disproportionally depending on the age of the contributing female and her oocyte quality [8], the importance of uterine receptivity [7] can also not be overlooked. Centers, which treat patients with more prior IVF failures, may, therefore, also be disadvantaged in oocyte donation cycles. Yet, devising a national reporting system which just has to exclude directed donor cycles and adjust for prior IVF cycles, appears technically much more achievable than a system which has to adjust for the myriad of covariates a general infertility population reflects.

Here demonstrated statistical correlation between center-specific IVF outcomes in donor and autologous cycles, therefore, can be seen as a first building stone in developing a fair, yet functionally more reliable reporting system for IVF cycle outcomes. This correlation, however, also allows for the establishment of “outliers”, which can serve centers as an internal quality of care parameter, leading them to a better understanding of where potential quality of care problems may be located at their center.

With expectations being that donor and autologous LBR correlate, centers that do not demonstrate such correlation should be able to deduct problem areas. For example, a center with donor LBR at national median but autologous LBR at only national 10^th^ percentile, can be, likely, assured about the center’s laboratory performance but should start wondering about the center’s clinical management of autologous cycles, unless the center serves a highly unfavorable patient population.

At the other extreme, a center at national median in autologous cycles but at national 10^th^ percentile in donor cycles, one has to wonder about egg donor selection and/or about, possibly, having good autologous IVF cycle outcomes only because of careful patient selection or too generous embryo numbers transferred.

In an attempt to better understand the potential of here presented data for internal and external quality control, our center assessed outlier clinics, which do not demonstrate the here reported correlation between donor and autologous cycles in national reports. Our preliminary findings were recently presented [12] and will be subject to a subsequent full length report.

## Conclusions

Since center-specific donor and autologous IVF cycle outcomes correlate, and as donor cycles reflect fewer patient covariates, they represent a first comparable performance measure between centers, allowing for internal as well as external quality control.

## Abbreviations

ART: Assisted reproductive technology
CDC: Centers for disease control and prevention
DOR: Diminished ovarian reserve
IVF: In vitro fertilization
LBR: Live birth rates
SART: Society for assisted reproductive technology
NASS: U.S. National Assisted Reproductive Technology Surveillance System.
